# *Angiostrongylus cantonensis* (Nematode: Metastrongiloidea): In Vitro Cultivation of Infective Third-Stage Larvae to Fourth-Stage Larvae

**DOI:** 10.1371/journal.pone.0072084

**Published:** 2013-08-20

**Authors:** Rong-Jyh Lin, Jie-Wen He, Li-Yu Chung, June-Der Lee, Jiun-Jye Wang, Chuan-Min Yen

**Affiliations:** 1 Department of Parasitology, School of Medicine, College of Medicine, Kaohsiung Medical University, Kaohsiung, Taiwan; 2 Institute of Medicine, College of Medicine, Kaohsiung Medical University, Kaohsiung, Taiwan; Universidade Federal de Minas Gerais, Brazil

## Abstract

The present study to attempt to cultivate *Angiostrongylus cantonensis* from third-stage larvae (AcL3) to fourth-stage larvae (AcL4) *in vitro* in defined complete culture medium that contained with Minimum Essential Medium Eagle (MEM), supplemented amino acid (AA), amine (AM), fatty acid (FA), carbohydrate (CA) and 20% fetal calf serum (FCS) was successful. When AcL3 were cultured in the defined complete culture medium at 37°C in a 5% CO_2_ atmosphere, the larvae began to develop to AcL4 after 30 days of cultivation, and were enclosed within the sheaths of the third molts of the life cycle. Under these conditions, the larvae developed uniformly and reached to the fourth-stage 36 days. The morphology of AcL3 develop to AcL4 were recording and analyzing. Then comparison of *A. cantonensis* larval morphology and development between *in vitro* cultivation in defined complete culture medium and *in vivo* cultivation in infective BALB/c mice. The larvae that had been cultivated *in vitro* were smaller than AcL4 of infective BALB/c mice. However the AcL3 that were cultured using defined incomplete culture medium (MEM plus 20% FCS with AA+AM, FA, CA, AA+AM+FA, FA+CA, CA+AA+AM or not) did not adequately survive and develop. Accordingly, the inference is made that only the defined complete medium enable AcL3 develop to AcL4 *in vitro*. Some nematodes have been successfully cultured into mature worms but only a few researches have been made to cultivate *A. cantonensis in vitro.* The present study is the first to have succeeded in developing AcL3 to AcL4 by *in vitro* cultivation. Finally, the results of *in vitro* cultivation studies herein contribute to improving media for the effective development and growth of *A. cantonensis*. The gap in the *A. cantonensis* life cycle when the larvae are cultivated *in vitro* from third-stage larvae to fourth-stage larvae can thus be solved.

## Introduction

*Angiostrongylus cantonensis* (Nematode: Metastrongiloidea), a nematode parasite, lives in the rat pulmonary artery where it develops to sexual maturity [1], [2]. *A. cantonensis* is thought to be the primary causative pathogen of human eosinophilic meningitis or meningoencephalitis in Taiwan, Japan, Southeast Asia and the Pacific Islands [3]–[5]. This parasite has spread from its traditional endemic regions of the Pacific islands and Southeast Asia to the South Pacific, Africa, India, the Caribbean, and most recently, to Australia and North America, including the USA, Caribbean islands and Brazil [6]. Humans and other non-permissive hosts acquire *A. cantonensis* by consuming raw terrestrial freshwater snails and slugs, or transport hosts, such as freshwater prawns, frogs, fish, and planarians.

Adult worms of *A. cantonensis* live in the pulmonary arteries of rats. The females lay eggs that hatch, producing first-stage larvae of *A. cantonensis* (AcL1) in the terminal branches of the pulmonary arteries. These AcL1 migrate to the pharynx, are swallowed, and are passed in the feces. They penetrate, or are ingested by, an intermediate host (snail or slug). After two molts and 14 days, the *A. cantonensis* reach the infective third stage (AcL3), in which they can infect mammalian hosts and remain viable for a long time. When a mollusk is ingested by a rat, which is the definitive host, the AcL3 exsheath in the rat stomach, penetrate the intestinal tissues and are transported via the heart to the brain and spinal cord approximately 17 hours after infection. The larvae molt to the fourth stage (AcL4) after 7 days and to the young adult between 9 and 11 days. They move to the subarachnoid space, where they grow. Between 28 and 36 days after infection, the young adult worms migrate to the lungs and heart via the cerebral venous system. Once they reach the branches of the pulmonary artery, they grow rapidly and achieve sexual maturity; oviposition then occurs between 40 and 50 days [7]. In week one the eggs embryonate and then hatch in the lung parenchyma. Consequently, the AcL1 can be recovered from the feces of the rat approximately 45 days after initial infection. When nonpermissive hosts such as mice, guinea pigs, rabbits, rhesus monkeys or people are infected with this parasite, the worms migrate to the brain, but fail to develop to sexual maturity within the heart or lungs [3]. The larvae in the brain provoke a severe acute inflammatory reaction, which mostly involves eosinophihic polymorphonuclear leukocytes. Furthermore, granulomas are found wherever *A. cantonensis* die, generally on the surface of the cerebral or cerebellar hemispheres. The pathogenesis of central nervous system (CNS) lesions is probably caused by direct or mechanical damage by the parasite during its migration in the CNS and by the metabolic products or antigens that are released by both living and dead parasites.

The *in vitro* cultivation of *A. cantonensis* biomedical research is required for angiostrongyliasis, related immunological diagnosis, and both physiological and biomedicial research. Although some nematodes have been successfully cultured into mature worms *in vitro* [8]–[10], only a few studies of the *in vitro* cultivation of *A. cantonensis* have been published. In earlier studies, adult worms of *A. cantonensis* have been cultured in *vitro* in the medium NCTC 109 that was supplemented with rat, horse or calf serum [11]. When NCTC 109 culture medium was supplemented with horse, rat or calf serum, *A. cantonensis* egg embryonated and hatched successfully. In a follow-up, when AcL1 was cultured in the medium that consisted of various ratios of L-15, Chermin’s balanced salt solution (CBSS), tryptose phosphatebroth (TBP) and fetal calf serum (FCS), AcL1 developed into the AcL2 and AcL3. When a medium that comprised various ratios of Minimum Essential Medium Eagle (MEM), Waymouth’s medium MB 752/1 (WYM), NCTC media 135 (NCTC 135), RPMI-1640 medium (RPMI-1640), *A. cantonensis* in the early third stage (early AcL3) developed to the late third stage (late AcL3) and AcL4 developed to the young adult stage [12].

Recently, the life cycle of *A. cantonensis* was examined by *in vitro* cultivation using a chemically defined culture medium but up to now the life cycle of *A. cantonensis in vitro* cultured system was still not completely set up. The gap of the life cycle *in vitro* cultured system was still evident. However, the successful culturing of AcL3 to AcL4 in *vitro* has not been reported upon. Although Hata [12] reported that early AcL3 developed to late AcL3 when cultured using MEM, WYM, NCTC 135 and RPMI-1640, AcL3 did not develop to AcL4 in the above medium. To date, the preparation of AcL4 has been inconvenient and has not been very easy to collect. This investigation is the first to describe successful in *vitro* development of AcL3 to AcL4.

A system for the in *vitro* cultivation of *A. cantonensis* would provide another tool for studying the morphology, physiology, pathology, biochemistry and immunology of *A. cantonensis*. The goal is to develop an *in vitro* culture medium system that is more defined in its composition and simpler to prepare, to facilitate the development of AcL4 from AcL3. In this paper, the defined medium that was used, supplemented with 20% FCS allowed AcL3 to develop into AcL4 *in vitro* cultivation. Finally, the gaps of the *in vitro* cultivation system for *A. cantonensis* are eliminated to reduce the sacrifice of animals in physiological, biochemical and immunological studies of *A. cantonensis*.

## Materials and Methods

### Ethics Statement

The use of animals in this study was reviewed and permitted by the Committee for the Care and Use of Laboratory Animals, Kaohsiung Medical University and was handled according to the protocol statement (Approval Number: NSC84-2331-B037-045) from the National Science Council, Taiwan, consistent with Taiwanese laws. Male Wistar rats and male BALB/c mice, weighing 200±10 g and 20±1 g, respectively, were obtained from the National Laboratory Animal Breeding and Research Center (Taipei, Taiwan). They were raised in the Laboratory Animal Center with air conditioning (at 22±1°C with a relative humidity of 50±10%) and illumination control (with lights on between 7∶30 and 19∶30). Animals were allowed food and water *ad libitum*.

### Drugs and Chemicals

Penicillin and streptomycin and other cell culture reagents were obtained from Gibco BRL Life Technologies (Grand Island, NY). Minimum Essential Medium Eagle (MEM), amino acid (AA), amine (AM), fatty acid (FA), carbohydrate (CA), fetal calf serum (FCS) and pepsin were obtained from Sigma-Aldrich Chemical Co. (St. Louis, MO). Millipore discs with 0.22 µm pores were obtained from Millipore (Merck Millipore Headquarters, MA).

### Preparation of AcL3

The Taiwanese strain of *A. cantonensis* that was used in this study was originally isolated from naturally infected giant African snails (*Achatina fulica*) that were collected in Pingtung County in southern Taiwan. *A. cantonensis* was maintained at the authors’ laboratory by cycling through the planorbid snail (*Biomphalaria glabrata*) and Wistar rats. AcL3 within planorbid snail tissues were isolated using our previous method with slight modifications [13]. Briefly, (snail shells were digested in 0.6% pepsin-HCl solution (pH 2–3, 500 I.U. pepsin/g tissue) with continued magnetic stirring at room temperature for one hour. Following digestion, the snail tissue suspension was allowed to stand for 30 min and larvae in the sediment were observed under a microscope. The morphological criteria for AcL3 were a length of 422 to 525 mm, a width of 24 to 35 mm and the termination of the tail at a fine point [14], [15]. Larvae that exhibited the greatest activity and fast s-type movement were utilized in the experiments.

### Defined Complete Culture Medium

Minimum Essential Medium Eagle (MEM) was supplemented with amino acid (AA), amine (AM), fatty acid (FA), carbohydrate (CA), and fetal calf serum (FCS). Table 1 shows the concentrations of the added AA, AM, FA and CA. The concentrations of FCS were 0, 5, 10, 15 and 20%, respectively. All defined complete culture media, containing penicillin (100 U/ml) and streptomycin (50 µg/ml), were sterilized by filtration using a Millipore disc with a pore size of 0.22 µm (Merck Millipore Headquarters, MA).

**Table 1:** The supplement factors of defined complete culture medium.

Additive	Unit (10^−3^ mmol)
**Amino acid (AA)**	
Phosphoserine	4.1
Taurine	7.5
Citrulline	2.5
2-Amino butyric acid	3.4
Ornithine	5.9
**Amine (AM)**	
Phosphoethanolamine	5.3
**Fatty acid (FA)**	
Palmitic acid	3.1
Palmitoleic acid	0.3
Stearic acid	2.9
Oleic acid	2
Linoleic acid	0.5
**Carbohydrate (CA)**	
Lactate	17.2
Dextrose (Glucose)	2.4

### Defined Incomplete Culture Medium

After the defined complete culture media with five concentrations of FCS, as described above, were tested, the FCS concentration that maximized the rate of development of AcL3 was used as basal constituent in the preparation of the incomplete culture media. To evaluate the effect of extra-supplement nutrient factors on the development of AcL3 during *in vitro* cultivation, culture medium supplements were varied and their effects of the variation in the development of the larvae were observed (Table 2).

**Table 2:** Uncomplete culture medium condition.

No.	Uncomplete culture medium
**1.**	Amino acid (AA) only
**2.**	Amine (AM) only
**3.**	Fatty acid (FA) only
**4.**	Carbohydrate (CA) only
**5.**	AA+AM+FA
**6.**	AA+AM+CA
**7**	FA+CA

Amino acid (AA): Phosphoserin, Taurine, Citrulline, 2-amino butyric acidand and Ornithine.

Amine (AM): Phosphoethanolamine.

Fatty acid (FA): Palmitic acid, Palmitoleic acid, Stearic acid, Oleic acid and Linoleic acid.

Carbohydrate (CA): Lactate and Dextrose (Glucose).

All uncomplete culture medium contained 20% FCS.

### *In vitro* Cultivation of AcL3

AcL3 were obtained from the infected planorbid snail, as described above. They were collected using a glass dropper with a thin tip. The larvae were placed in a sterilized Eppendorf tube to which had been added sterilized phosphate buffered saline (PBS) that contained antibiotics. They were washed five times by centrifugation with 1000 rpm for 5 min at room temperature. The worms were then transferred into 24-well plates that contained 1 mL medium, and were incubated in a 5% CO_2_ atmosphere at 37°C. Each well contained 50 AcL3 in 1 mL medium, which was changed every 72 hours. Twenty larvae were randomly collected every 72 hours to examine their development, molting and survivability under an inverted microscope and a light microscope.

### Identification of Larvae Development

Larvae were identified using the following criteria, which were proposed by Hata *et al.*, [9]. AcL3 had a rod-shaped structure at the anterior end of the body. They exhibited fast s-type movement and had sharp tails. AcL4 were longer than AcL3 and were enclosed within the sheath of the fourth molt; the rod-shaped structure had disappeared. The movement of the larvae was different from that of AcL3 and they had blunt tails.

### Measurement of Size of Larvae of *A. cantonensis*

Each BALB/c mouse was infected with 50 AcL3. Two mice were daily sacrificed until seven days after infection. Twenty larvae were collected daily from the brain of two infected mice after infection. An image of each cultivated larva and each larva from infected mice was used to measure the length and width using analytical Imaging Station™ software (Imaging Research Inc, St. Catharines, Ontario, Canada).

### Statistical Analysis of Data

The results are presented as mean ± SD. Statistical differences were estimated by one-way analysis of variance (ANOVA) followed by Student’s *t* test. A p value of 0.05 was considered to be indicate significance. Data were analyzed and figures plotted using appropriate software (Sigma Plot Version 8.0 and Sigma Stat Version 2.03, Chicago, IL) run on computer.

## Results

### Cultivation of AcL3 in Complete Culture Medium

**Figure 1:**
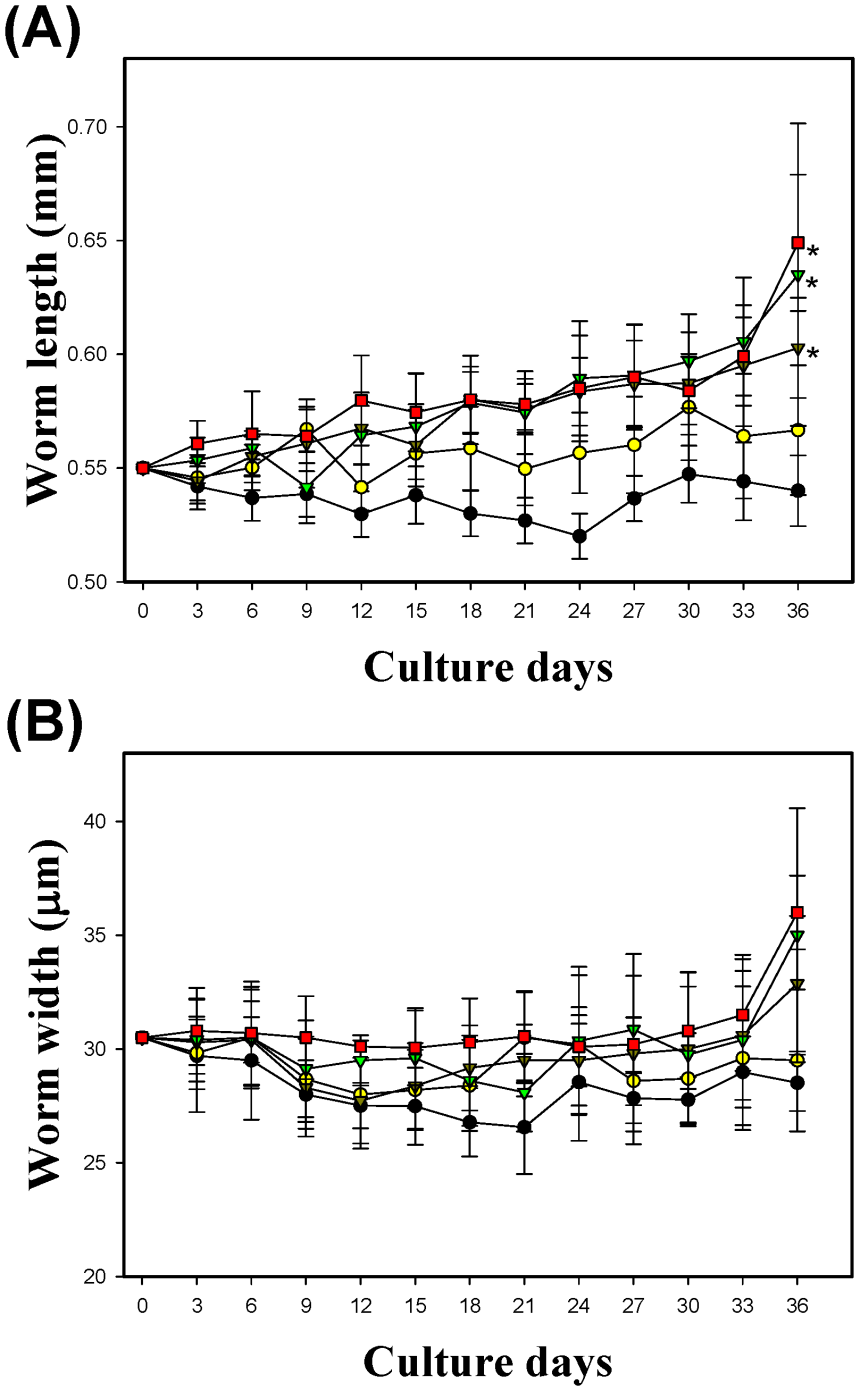
Changes of body length (A) and width (B) in third-stage larvae after culture with complete medium that contained 0 to 20% FCS. All worm body lengths on day 36 after culture with complete medium that contained FCS significantly exceeded those of third-stage larvae before culture. (*p<0.01, **p<0.05).

The variations in AcL3 length and width over time were examined. During the culturing, the length of AcL3 increased significantly with time (Fig. 1A), but the width did not (Fig. 1B). With 10%, 15% and 20% fetal calf serum (FCS), (Fig. 1A and 1B), AcL3 were significantly longer on day 36 than on day 0 (initial). The AcL3 were cultured in various chemically defined complete culture media (Table 1) with 20% FCS for 21–36 days.

As indicated in Table 3, none of the AcL3 had developed to AcL4 on day 21, 24 and 27. However, as the culture duration increased, the percentage of larvae in the AcL4 stage of development increased, while that in the early AcL3 stage decreased. On day 36, the percentage of AcL4 exceeded all earlier days.

**Table 3:** The development of larvae cultured with complete medium containing 20% FCS.

Cultureddays	Percentage of development				Total NO.of worms
	AcL3			AcL4	
	Early	Middle	Late		
21	88	13	0	0	24
24	51	43	6	0	53
27	45	23	32	0	22
30	39	47	14	2	24
33	25	38	29	8	51
36	11	15	33	41	27

AcL3 were isolated from planorbid snail tissues and detailed methods can be found in “Preparation of AcL3” of Materials and methods section.

**Figure 2:**
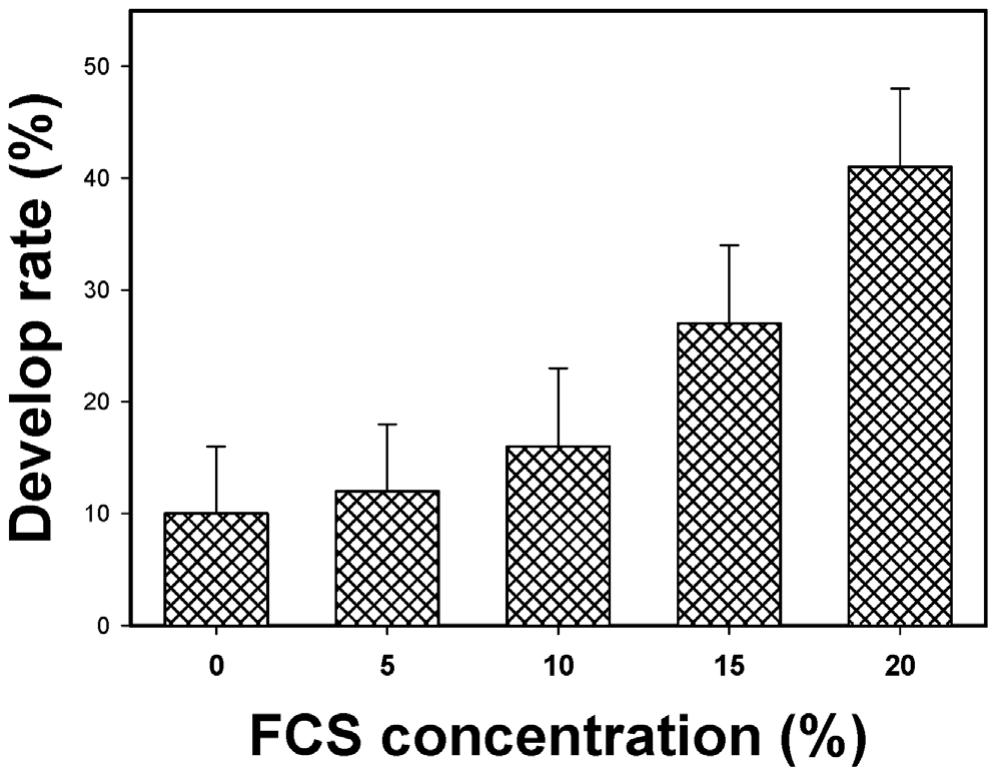
Rates of development of third-stage to fourth-stage larvae after 36 days of culture in the complete medium that contained different concentrations of FCS. The rates of development are determined percentage of fourth-stage larvae numbers of all cultured worms.

FCS is an important component of the defined complete culture medium. Therefore, the FCS concentration during cultivation was varied to evaluate its effect on the rate of development of larvae. Moreover, when AcL3 were cultured in complete culture medium plus 5%, 10%, 15% and 20% FCS, the rate of development of AcL3 to AcL4 with molting increased with the concentration of FCS (Fig. 2). The complete culture media with 20%, 15% and 10% FCS provided higher rates of development than the media with 5% and 0% FCS. The complete culture medium with 20% FCS was associated with the fastest development. Hence, 20% FCS was used in the basal medium condition in the preparation of the aforementioned incomplete culture medium.

### Cultivation of AcL3 in Incomplete Culture Medium

**Figure 3:**
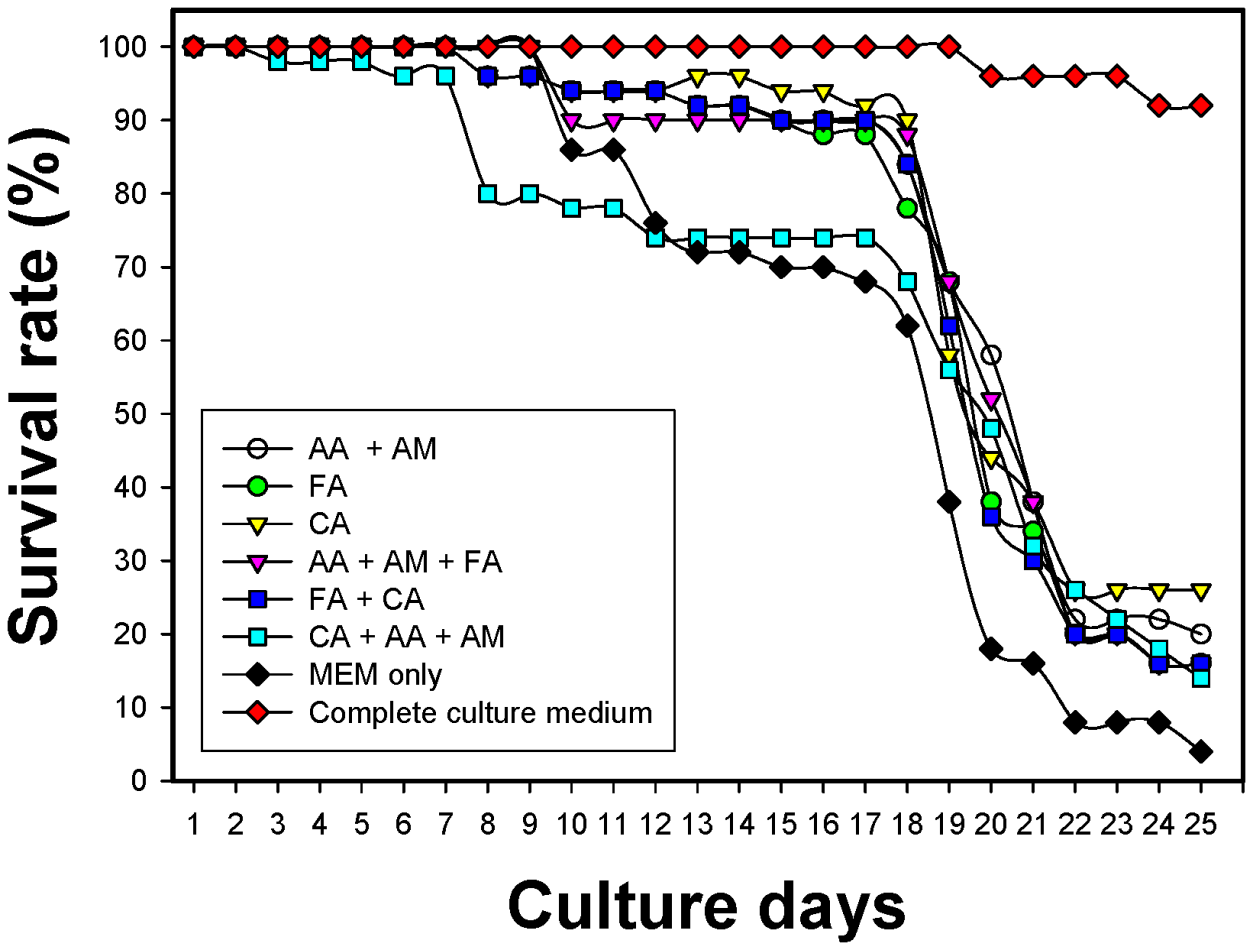
Survival rates of AcL3 after culture with complete and incomplete media that contained 20% FCS. The survival rates are definitived as 100% on culture day 1 and follow determined as culture day.

A 20% proportion of FCS provided the optimal culture condition, which was used in the incomplete culture medium in the following steps. The survival rates of AcL3 after culturing with Minimum Essential Medium Eagle (MEM) plus 20% FCS and another other supplements were determined. Each medium was supplemented with amino acid (AA), amine (AM), fatty acid (FA) and/or carbohydrate (CA), as shown in Fig. 3. The culture results demonstrate that AcL3 did not have a high survival rate when the above one or two chemical supplements were added to the culture medium. Before day eight, all culture media plus AA, AM, FA and/or CA, was associated with a high survival rate under all conditions. After day 10, the survival rate of AcL3 declined slowly in all incomplete culture media. On day 20, many of the AcL3 were dead, except in the complete culture medium which contained MEM, 20% FCS, AA, AM, FA and CA. AcL3 had a high survival rate of above 90% in the complete culture medium. None of the incomplete culture media supported any larval development (data not shown).

### Morphology of AcL3 and AcL4 in Complete Culture Medium

**Figure 4:**
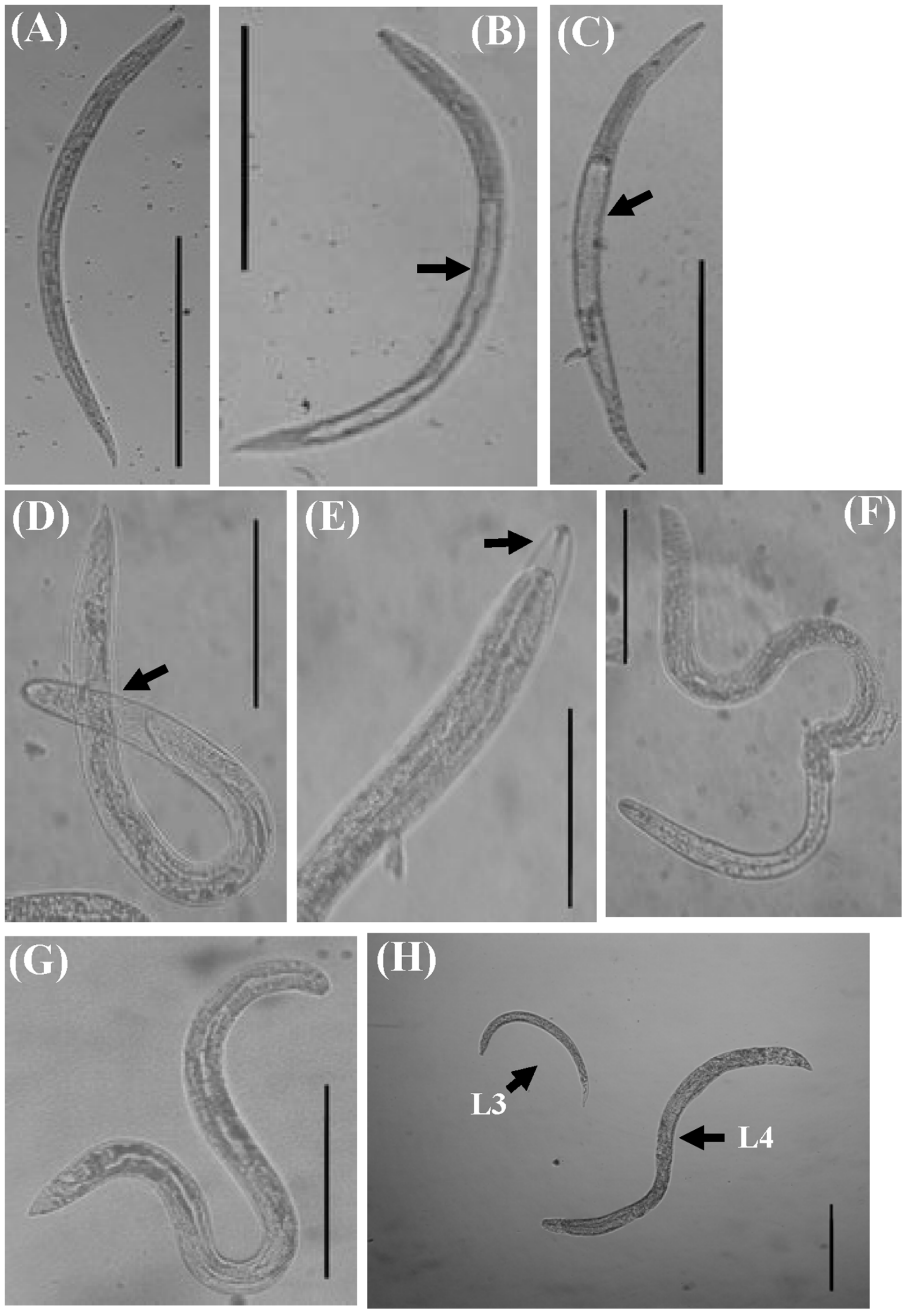
Larvae of *A. cantonensis* developed in complete medium that contained 20% FCS. (a) Early third-stage larva. (b) Middle third-stage larva, showing evacuation of gut (arrow). (c) Late third-stage larva, showing expansion and evacuation of gut (arrow). (d) Fourth-stage larva enclosed in sheath (arrow). (e) Anterior end of fourth-stage larva, showing rod-shaped structures retained in sheath (arrow). (f) Exsheathing fourth-stage larva. (g) Exsheathed fourth-stage larva. (h) Comparison of sizes of fourth-stage larva from *in vitro* culture and third-stage larva of *A. cantonensis*. Fourth-stage larva on day 36 of culture (lower right-hand side) is significantly larger than third-stage larva (upper left-hand side). Bar = 0.25 mm.

Figure 4 display the morphology of larval *A. cantonensis* that had developed in the complete culture medium that contained 20% FCS. Figure 4A shows an early third-stage larva. Fig. 4B shows a middle third-stage larva, showing the evacuation of the gut (arrow). Fig. 4C shows a late third-stage larva, showing the expansion and evacuation of the gut (arrow); Fig. 3D shows a fourth-stage larva, enclosed in a sheath (arrow); Fig. 4E shows anterior end of a fourth-stage larva, showing the rod-shaped structures that are retained in the sheath (arrow); Fig. 4F shows an exsheathing fourth-stage larva, and Fig. 4G shows an exsheathed fourth-stage larva (Fig. 4G). After the larvae had been cultured for 30 days, they were completely surrounded with sheaths, and the rod-shaped structures, characteristic of AcL3, were retained in the anterior sheaths. On day 32 of the culture, the observed space between the anterior sheath and the buccal cavity of AcL4 was larger than on day 30 and the rod-shaped structure at the anterior sheath and blunt tail had disappeared. On day 33, the larvae swing their tails to molt their sheath. The last half of the sheath was molted from the tail part of the larva on day 34. Finally, almost all of sheath had molted from the larva on day 35. In addition to molting in the development of AcL4, the movement of the larva changed, becoming slower than that of AcL3. AcL4 were also longer than AcL3. In Fig. 4H, AcL4 on day 36 culture in complete medium that contained 20% FCS (lower right-hand side) were significantly larger than AcL3 (upper left-hand side). Although AcL3 molted to become AcL4, the larvae could not develop to young adult and died.

### Comparison of Development of *A. cantonensis* Larvae in Incomplete Culture and Complete Culture Media

Table 3 presents the larval development in complete culture medium plus 20% FCS. The larvae molted to late L3 on day 24 of the culture; L4 with enclosed sheaths were observed on day 30 of culture. Only in the MEM culture medium without any added AA, AM, FA or CA supplement was the larval survival rate lower than in the incomplete culture medium (Fig. 3) and no AcL3 developed to AcL4 (data not shown).

### Comparison of *A. cantonensis* Larval Morphology and Development between *in vitro* Cultivation in Medium and *in vivo* Cultivation in Infective BALB/c Mice

**Figure 5:**
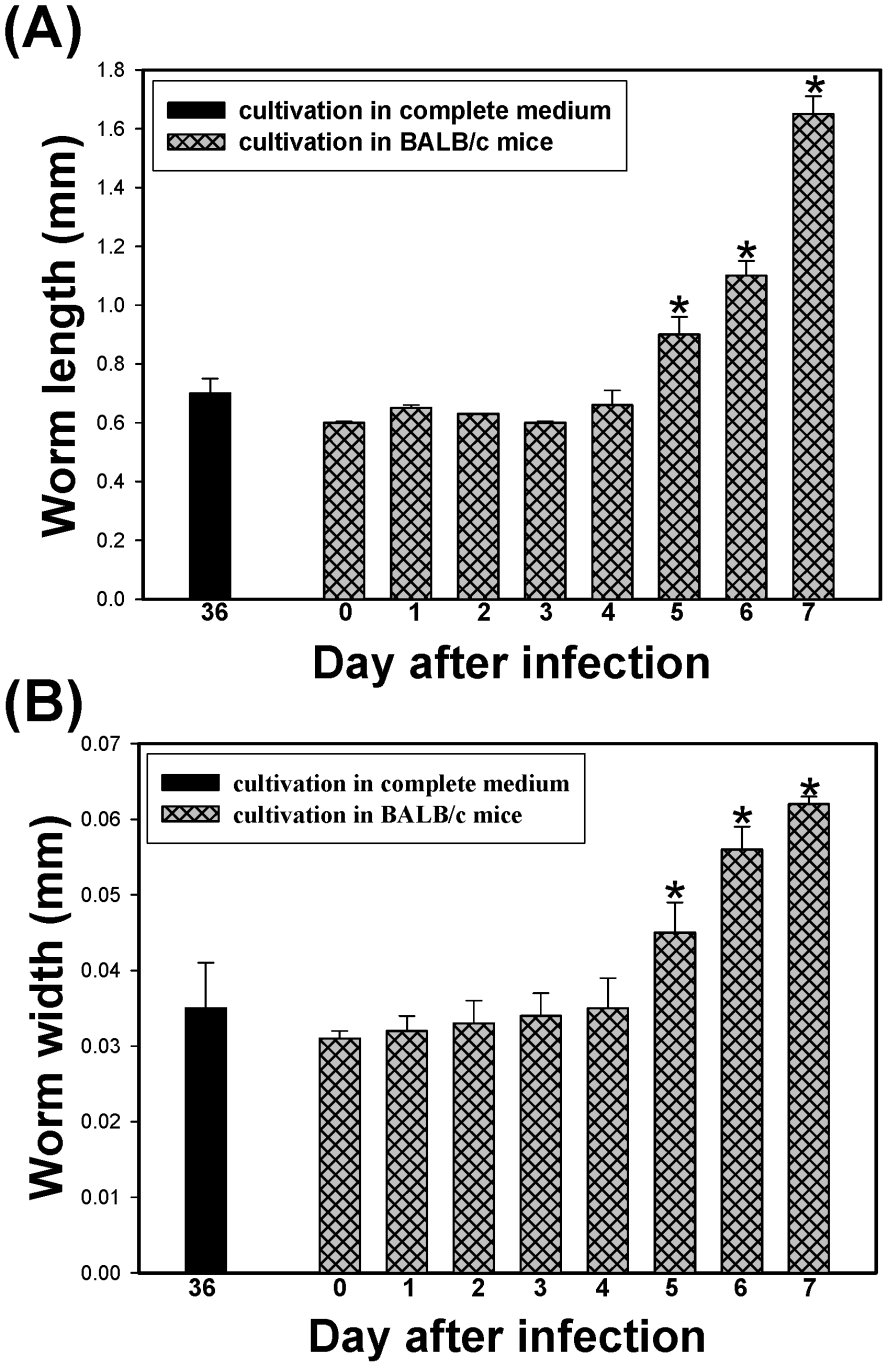
Comparisons of length (5A) and width (5B) of worms 36 days after culture with complete medium that contained 20% FCS with those of worms from brain of BALB/c mice after infection with *A. cantonensis*. Both length and width of worms from brain of BALB/c five days after infection were significantly larger than those of worms cultivated *in vitro* (*p<0.01).

*A. cantonensis* larvae developed *in vivo* by cultivation in infective BALB/c mice (Fig. 5). As the infection time increased, the development of *A. cantonensis* larvae proceeded and mature AcL4 were observed on day 5 of culturing. Figure 5 compares the worms that developed *in vivo* in infective BALB/c mice on day 0–7 with those that developed *in vitro* in complete medium that contained 20% FCS on day 36. The mean length (Fig. 5A) and width (Fig. 5B) of *A. cantonensis* worms that developed *in vivo* exceeded those *in vitro*. Although this study is the first successfully to develop AcL3 to AcL4 by *in vitro* cultivation in complete medium, the size of the cultured worms was not the same as that of the worms that were cultivated *in vivo* in infective BALB/c mice. AcL3 was observed before day 4 of infection. However, AcL4 was observed on days 5, 6 and 7 following infection. The development of AcL3 to AcL4 took 30 days with *in vitro* cultivation, but only five days with *in vivo* cultivation in the brains of mice. The worms in the former case were significantly smaller than in the latter.

## Discussion

Some nematodes have been successfully cultured into mature worms but only a few attempts have been made to cultivate *A. cantonensis in vitro* [16]–[18]. Some attempts have been made to maintain adult worms *in vitro* and to obtain AcL1 by the culturing of deposited eggs [19]. Some researchers have successfully cultured *A. cantonensis* from the first-stage to infective third-stage larvae [20]. Subsequently, fourth-stage larvae of *A. cantonensis* were successfully cultured to the young adult stage [12]. However, no report on the *in vitro* cultivation of *A. cantonensis* from the third stage (AcL3) to the fourth stage (AcL4) has been published. Therefore, this study presents successful attempts to culture theAcL3 to AcL4 in chemically defined media. In this study, *A. cantonensis* was successfully cultured *in vitro* from the infective third-stage to fourth-stage larvae, which are known to live in the host’s brain.

The mean body length, width and development time of AcL4 that were cultivated *in vitro* were shorter, narrower and longer, respectively, than those that developed *in vivo* in mice brains, and the percentage development *in vitro* was only 41±5%, which is much lower than is seen in mice brains.

In *vitro* cultivated AcL3 molted around day 30 to AcL4. In this culture stage, the rod-shaped structure of AcL3 was observed in the cast-off cuticle. After the old cuticle had been cast off, the larvae moved in a different way from the AcL3. The AcL4 exhibited lower mobility with slower and longer oscillation than were common for AcL3. The long time of the development of the cultured AcL3 to AcL4 was observed as expected, since the growth and development of parasitic helminthes *in vitro* is known to be slower than those *in vivo,* and the former cultured worms were also smaller [8]–[10].

When AcL3 were cultured in the incomplete culture medium MEM without any supplement, the larval survival rate declined with time. In the complete culture medium, on the other hand, we observed a high larval survival rate. After approximately 20 days of culturing, the gut of AcL3 that were cultivated *in vitro* began to expand and their body width slightly increased. These larvae could not develop to AcL4 when *in vitro* cultivation, but died because the incomplete culture medium lacked indispensable supplements.

In previous investigations, the most effective medium for the development of *A. cantonensis* was a chemically defined [11], [12], [19]–[22]. For example, RPMI-1640, WYM and Ham’s F-12 media were designed for the culturing of mammalian cells. Although some parasitic nematodes of vertebrates have been successfully cultured to maturity, AcL3 had not been reported to have been cultured to AcL4 in a chemically defined medium before our study.

In earlier studies, the *in vitro* cultivation of different stages of *A. cantonensis* successfully developed because criteria for various life-cycle stages were identified [11], [12], [19], [20], [22]. AcL1 has been developed to AcL3 only in low-osmotic media, because the most important characteristic of snail hemolymphs is their low osmolarity. Weinstein *et al.* [11] reported that when AcL1 were cultured in various media which is used for culture mammalian cells previously, but no growth or differentiation occurred in any of the media that they used. Therefore, low osmolality may be one of the most important factors in the development of larvae of *A. cantonensis* [20].

Furthermore, Hata et al. [12] demonstrated that morphological and developmental of AcL3 and AcL4 in various media were different, respectively. AcL3 have been cultured in various chemically defined media without a serum supplement for four weeks, and 9% of the worms in RPMI-1640 and 4% of the worms in NCTC 135 developed to the late third stage in WYM and Eagle’s MEM. However, when these defined media were supplemented with 20% fetal bovine serum (FBS), larval development was observed in all media. In particular, in RPMI-1640 that was supplemented with 20% FBS, more than 30% of the worms developed to the late third stage, although they gradually died subsequently. Unfortunately, in the above investigation, AcL3 failed to develop to AcL4. Although the present study is the first to have succeeded in developing AcL3 to AcL4 by *in vitro* cultivation in a complete medium, the cultured worms were not the same size as those that were cultivated *in vivo* in *A. cantonensis* infected BALB/c mice.

In the cultivation of *A. cantonensis* herein, AcL3 successfully developed to AcL4, but they did not develop to young adult as they do in rat brain; rather, the larvae slowly died as the culture time increased. Accordingly, in the attempt herein to develop AcL3 to AcL4, first, some of the environmental conditions under which normal AcL4 live in their final host were mimicked. In fact, when the definitive host ingests infective AcL3, the AcL3 exsheaths in the stomach; penetrate the intestinal tissues, and are carried by the venules to the lungs and other tissues throughout the body. Then, the larvae migrate to the brain and congregate in the spinal cord, cerebellum, medulla, and diencephalon, whence they slowly spread to the telencephalon and migrate into the subarachnoid space where they moult twice to become AcL4 and then young adult. Based on this actual life cycle, we suggest that the brain environment is important to develop AcL3 to AcL4. Finally, according the component of brain cells/tissue culture medium and the constituents of cerebral spinal fluids (CSF), MEM was used as the basal complete culture medium for *in vitro* cultivation.

The MEM was supplemented with AA, FA and/or CA, which are the constituents of CSF [23]–[27]. MEM is usually used to culture brain cells. They are used in *in vitro* cultivation studies to promote the development and growth of *A. cantonensis*. Finally, the results of *in vitro* cultivation studies herein contribute to improving media for the effective development and growth of *A. cantonensis*. The gap in the *A. cantonensis* life cycle when the larvae are cultivated *in vitro* from AcL3 to AcL4 can thus be solved.
